# Cl^−^, Na^+^ and Mg^2+^ Adsorption and Electronic Properties on 2-Octyl Acrylate and Isobornyl Acrylate Monomers: A Comprehensive DFT Study

**DOI:** 10.3390/polym17060799

**Published:** 2025-03-18

**Authors:** Emre Bolen, Jorge S. Dolado, Andrés Ayuela

**Affiliations:** 1 Centro de Fisica Materiales, CFM-Material Physics Center (MPC), CSIC-UPV/EHU, Paseo Manuel Lardizábal 5, 20018 Donostia-San Sebastian, Spain; j.dolado@ehu.eus; 2 Donostia International Physics Center (DIPC), Paseo Manual Lardizábal 4, 20018 Donostia-San Sebastian, Spain; 3 Opticianry Program, Department of Medical Services and Techniques, Vocational School Health Services, Aksaray University, 68100 Aksaray, Türkiye

**Keywords:** acrylate monomers, ion adsorption, electronic properties, density functional theory calculations

## Abstract

The design of advanced functional materials from polymers involving 2-octyl acrylate and isobornyl acrylate monomers is crucial for applications such as biofouling resistance, coatings, UV-curable films, and use in marine environments. In this study, we investigated the adsorption and electronic properties of 2-octyl acrylate and isobornyl acrylate monomers in the presence of Cl^−^, Na^+^, and Mg^2+^ ions using Density Functional Theory calculations. Adsorption energies, quantum descriptors, and electrostatic potential maps were analyzed to elucidate ion-specific interactions with these monomers. Our findings indicate that Mg^2+^ ions exhibit the strongest interactions due to their high charge density, followed by Na^+^ and Cl^−^ ions, which show moderate and weak adsorption, respectively. Density of states analyses revealed that Mg^2+^ significantly lowers HOMO and LUMO levels, narrowing the gap and stabilizing the system, while Cl^−^ ions result in a smaller gap and weaker interactions. Electrostatic potential maps further confirmed these trends, correlating ion adsorption sites with molecular charge distributions. This study highlights the critical role of ion adsorption and its associated electronic properties and paves the way for future advancements in optimizing 2-octyl acrylate and isobornyl acrylate-based materials for applications such as coatings and use in marine environments.

## 1. Introduction

The marine industry widely employs polymers and composite materials in applications such as renewable marine energy systems, offshore oil and gas infrastructure, glass fibers, coatings, etc. [1,2]; however, these materials must also contend with hazardous effects [3] on marine environments, including chemical attacks, biofouling [4], and similar threats. Therefore, factors such as resistance to chemical attacks, recyclability, moisture absorption, adequate adhesion, biofouling, etc., should be carefully considered when designing polymer-based materials for harsh marine applications.

In the field of polymer materials, acrylate monomers have recently come to the forefront in marine environments, particularly in terms of dealing with biofouling [4]. Therefore, acrylate-based monomers are already widely investigated for applications in marine environments [4,5,6]. Moreover, acrylate monomers exhibit a wide range of properties, including superior absorbency, optical transparency, mechanical flexibility, as well as enhanced toughness and hardness [7]. These properties make them highly versatile for applications ranging from biomedical fields [8] to marine coatings. Among the acrylate monomers, 2-octyl acrylate (2-OA) stands out as a bio-based monomer of significant industrial relevance, due to its established large-scale production [9], commercial availability, and broad applicability across multiple industrial sectors [10]. In addition to 2-OA, this study also considers isobornyl acrylate (IBOA), a monomer derived from natural, renewable turpentine oil [11,12]. IBOA is widely used in the production of UV resins [13], hydrogels [14], polyacrylates [15], and polyurethanes [16], as well as in consumer products such as adhesives, sealants, inks, plastics, rubber, and coatings [17], and in medical devices such as glucose sensors [18]. Furthermore, mixed 2-OA and IBOA monomers have been investigated for the development of advanced functional polymers, including water-borne coatings, pressure-sensitive adhesives, and UV-curable films [19,20,21].

Adsorption is a critical process for understanding the fundamental surface properties of materials and plays a key role in fields ranging from materials engineering and catalysis to environmental science and energy storage, where surface interactions are essential for performance and efficiency. For instance, the adsorption energy has a direct impact on monomer–substrate interactions, catalysis [22], the polymerization process, as well as the thermodynamic properties [23], and thus plays a dominant role in the potential applications of monomers and dimers. Specifically, the interactions of metal cations (Na^+^, Mg^2+^) and anions (Cl^−^) with organic molecules, such as 2-OA and IBOA monomers and dimers, are key for tailoring their electronic and surface properties for specific applications, such as coatings, nanocomposites, and polymer systems. More specifically, these monomers play a key role, particularly in surface coatings in marine environments due to chemical attacks with Cl^−^, Na^+^, and Mg^2+^ ions typically found in salty waters.

Density Functional Theory (DFT) [24,25], known for its capability of performing first-principles calculations, has emerged as an indispensable tool for its accurate prediction of not only the electronic and structural properties, but also the adsorption behavior of complex materials. DFT has been extensively applied to investigate adsorption mechanisms [26] and to identify functional monomers [27]. For example, previous studies [28] have utilized DFT calculations to examine adsorption phenomena such as inhibitor adsorption on montmorillonite surfaces [28] or monomer adsorption to design novel flocculants [29].

In this study, we employ DFT to explore the electronic properties and adsorption mechanisms of Cl^−^, Na^+^, and Mg^2+^ ions on both 2-OA and IBOA monomers. By analyzing the adsorption energy, charge distribution, and electronic properties of these systems, we aim to establish a comprehensive understanding of their surface chemistry and its potential applications. The findings from this study will help in the design and optimization of polymer-based materials; based on surface-specific interactions and ion adsorption properties we will optimize applications in coatings and marine environments.

## 2. Computational Details

DFT-based calculations were performed using the SIESTA code [30,31] using split-type double (DZP) basis sets [32]. Calculations were performed using generalized gradient approximation (GGA) with the Perdew–Burke–Ernzerhof (PBE) exchange–correlation functional [33]. A standard cutoff energy of 100 meV for confining orbitals was used in all calculations, and a large energy cutoff of 700 Ry was considered. The conjugate gradient (CG) method [34] was used for atomic relaxations, allowing the atoms to fully relax until the forces were below 0.01 eV/Å for the pure structures and less than 0.02 eV/Å for monomers with adsorbed ions. The Γ point was used for [35] k-point sampling when performing relaxations and electronic structure calculations. A separation distance of about 20 Å between the replicated monomers in adjacent cells along the three axes was used to eliminate intercell interactions.

After relaxing the atoms, the adsorption energy $E_{ads}$ was calculated using the following equation,

(1) $$E_{ads}=E_{adsorbed}^{mononer/dimer}-E_{pure}^{mononer/dimer}-E_{ion}$$

where $E_{adsorbed}^{mononer/dimer}$ is the total energy of the ion-adsorbed monomer or dimer, $E_{pure}^{mononer/dimer}$ is the total energy of the pure monomer or dimer, and $E_{ion}$ is the total energy of Cl^−^, Na^+^ or Mg^2+^ ions.

Finally, we analyzed the most energetically favorable adsorption configurations and calculated quantum molecular descriptors, such as the chemical potential (*μ*), the global hardness (*η*), the electrophilicity index (*ω*), as well as the HOMO-LUMO energy levels and gaps. By a finite difference approach [36], the chemical potential and the global hardness were calculated as,

(2) $$\;\mu =-\frac{1}{2}\left(I+A\right),\;\eta =\frac{1}{2}(I-A)\mathrm{and}\;\omega =\frac{\mu^{2}}{2\eta }$$

where *I* is the ionization potential, and *A* is the electron affinity. Furthermore, using Koopman’s theorem [37,38], the ionization potential (*I*) and electron affinity (*A*) values were derived from the HOMO and LUMO energy levels using the frontier orbitals, as expressed by the following equation,

(3) $$I=-E_{HOMO}\;\mathrm{and}\;A=-E_{LUMO}$$

These calculations provide a detailed understanding of the electronic and adsorption properties and offer insights into the ion-specific interactions with the monomers.

## 3. Results and Discussions

### 3.1. Adsorption Energy and Quantitative Analysis

**Figure 1:**
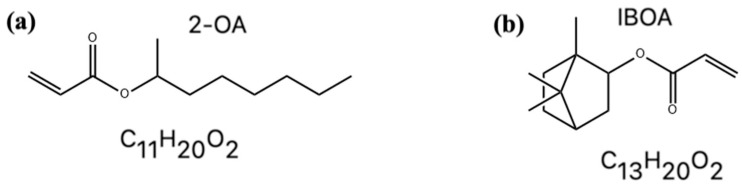
Chemical structure of two acrylate monomers: (**a**) 2-octyl acrylate and (**b**) isobornyl acrylate.

The chemical formula of the acrylate monomers 2-OA, C_11_H_20_O_2_ has eleven carbon atoms, twenty hydrogen atoms, and two oxygen atoms in a linear structure. The IBOA has a more complex structure, with a chemical formula C_13_H_20_O_2_ that reflects a similar atomic composition to 2-OA, but with two additional carbon atoms. It also has a more branched and ring-like structure. Figure 1 represents the chemical structures and chemical formulas of the two the acrylate monomers 2-OA and IBOA, often employed in various industrial and biomedical applications due to their desirable properties.

**Figure 2:**
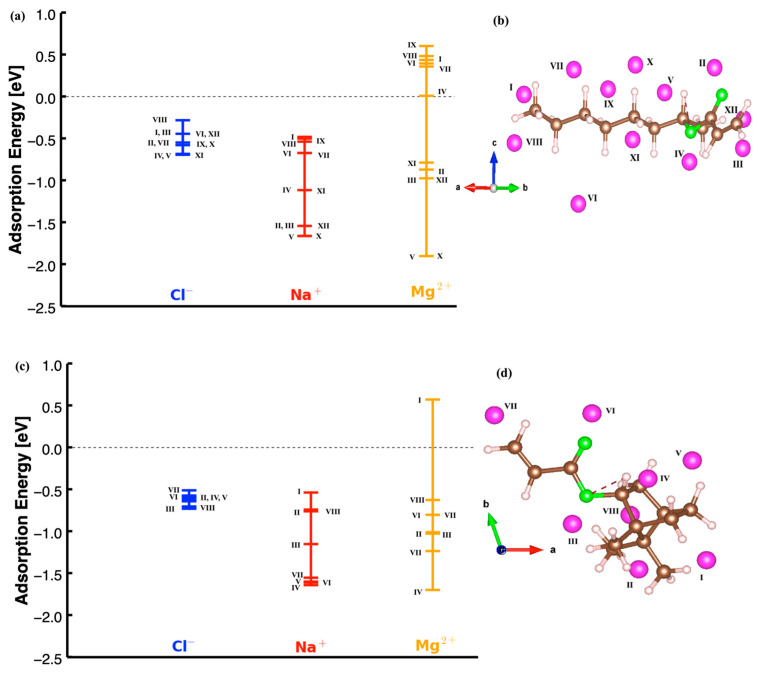
(**a**) Distribution of adsorption energies and (**b**) input configurations for Cl^−^, Na^+^, and Mg^2+^ ions across different sites on 2-OA. (**c**) Distribution of adsorption energies and (**d**) input configurations for Cl^−^, Na^+^, and Mg^2+^ ions across different sites on IBOA. The pink-colored spheres denote adsorbed ions (Cl^−^, Na^+^, Mg^2+^) given by roman numbers; the green, brown, and white spheres denote oxygen, carbon, and hydrogen atoms, respectively.

The chemical structure of 2-OA consists of a linear chain with an acrylate group at one end, as shown in Figure 1. The acrylate group is responsible for its reactivity, particularly in polymerization processes. The IBOA structure is bicyclic compared to the 2-OA one, with the characteristic isobornyl group attached to the acrylate moiety. After obtaining the relaxed structures for both monomers, Cl^−^, Na^+^, and Mg^2+^ ions were placed at multiple positions to calculate their adsorption energies and identify the most energetically favorable configurations. Figure 2 shows the results obtained for 2-OA and IBOA, including the positions where the ions were placed as inputs.

#### 3.1.1. Adsorption Energy Trends

Figure 2 shows the dispersion of the adsorption energies (in eV) for different ions—Cl^−^ (in blue), Na^+^ (in red), and Mg^2+^ (in orange)—at several initial positions on 2-OA and IBOA which are indicated by Roman numerals on the structures shown on the right-hand side. For the different ions, the *y*-axis gives the adsorption energy in Figure 2a,c. The calculated results for 2-OA, illustrated in Figure 2a, show negative adsorption energy values, indicative of exothermic adsorption processes. The adsorption energy values for Cl^−^ are clustered near 0 eV, and are relatively weak adsorption interactions, with most values being slightly negative, reflecting a favorable but weak binding. For 2-OA, the lowest adsorption energies of Cl^−^ are observed for the positions IV, XI, and V, with values of −0.68, −0.69, and −0.69 eV, respectively, suggesting that these positions are more energetically favorable for Cl^−^ binding compared to other sites. Other positions, such as I, III, II, and VII, show values ranging from −0.55 to −0.44 eV, with a relatively weaker adsorption. The position VIII, with an adsorption energy of −0.28 eV, has the lowest adsorption energy compared to other positions, and it is the least energetically favorable site.

The Na^+^ ions show a wider range of adsorption energies compared to Cl^−^, with several sites exhibiting even more negative energies, which indicate stronger adsorption interactions. The most stable adsorption occurs at the positions V and X, with adsorption energies of about −1.66 eV, which are the lowest among all sites, indicating that these sites are particularly favorable for Na^+^ adsorption. The positions II, III, and XI show adsorption energies close to −1.54 eV, a value which is also indicating strong adsorption, although weaker than the one at position V. Some positions, such as VI, VII, and VIII, are clustered in the range between −0.5 and −0.67 eV, and show much weaker adsorption interactions, like for Cl^−^ ion. For the Na^+^ ion, the least energetically favorable position is the position I with an adsorption energy of −0.48 eV, and the difference between this one and the most energetically favorable position is about 1.2 eV.

The Mg^2+^ ions, being doubly charged, exhibit a wider range of adsorption energies, which includes positive adsorption energies, unlike for Na^+^ and Cl^−^. The most stable adsorption is observed at positions V and X, with energies around −1.90 eV, the lowest among all ions and positions because Mg^2+^ binds much more strongly to these sites. The positions II, III, XI, and XII exhibit energy ranges between −0.97 and 0.79 eV and indicate strong adsorption interactions at these sites as well. In addition, for the Mg^2+^ ion, there are positive adsorption energies observed at positions I, IV, VI, VII, VIII, and IX, with values of 0.44, 0.01, 0.40, 0.36, 0.48, and 0.61 eV, respectively. These positive energies, not observed for Cl^−^ and Na^+^, indicate that the adsorption mechanism for the Mg^2+^ ion is highly dependent on its localization on the 2-OA structure. The energy difference between the highest positive adsorption state and the lowest negative energy adsorption is about 2.5 eV. For the Mg^2+^ ion, the adsorption energy becomes positive with increasing distances from the oxygen atoms, a fact that also suggests much weaker interactions. When considering all three ions (Cl^−^, Na^+^, and Mg^2+^), position V consistently exhibits the lowest adsorption energy, making it the most energetically favorable site for adsorption across all three ions.

The calculated adsorption energies for the IBOA monomer for each ion are shown in Figure 2c, and the corresponding adsorption positions are given in Figure 2d. Similarly to 2-OA, it is observed that for IBOA, the adsorption energy range and values for the Cl^−^ ion are significantly lower compared to the other two ions when evaluated together. Moreover, the adsorption energy for the Cl^−^ ion varies between approximately −0.5 eV and −0.7 eV, within a range of 0.2 eV. This suggests that the adsorption energy of the Cl^−^ ion is relatively independent of its specific location on the more 3D structure of the IBOA monomer. In contrast, the sodium ion (Na^+^) exhibits stronger adsorption at most positions, and the lowest adsorption energies for Na^+^ are observed at positions IV, V, and VI, which highlight that these sites are energetically favorable for Na^+^ binding. Moreover, for the Na^+^ ion, the lowest energy configuration is at site IV with −1.64 eV, while the highest energy configuration is at site I with −0.54 eV, with a difference of approximately 1.1 eV. The change in the adsorption energies for Na^+^ across different sites suggests that the interaction strength is more dependent on the specific location compared to Cl^−^ ions. The magnesium ion (Mg^2+^) shows the most stable adsorption energies, with values reaching much lower (more negative) than those for the Cl^−^ and Na^+^ ion. This indicates that Mg^2+^ binds much more strongly to the IBOA monomer due to its higher charge density. Unlike the other two ions, the Mg^2+^ ion exhibits a positive adsorption energy of about 0.6 eV at site I. We observed that this position is generally surrounded by a high density of H atoms, so that the Mg^2+^ ion, having lost two electrons, is destabilized due to interactions with H bonds. Moreover, the adsorption energies for Mg^2+^ vary across different sites, with the most favorable binding occurring at position IV with the lowest adsorption energy value of −1.70 eV. In addition, for the Mg^2+^ ion, the adsorption energy values range between −1.2 and 0.7 eV for positions I and IV, respectively. Note that transition metal ions would behave like Mg^2+^ ions and are expected to have strong adsorption energies due to their positive charges as well as the 3D electrons, which could form even stronger interactions with 2OA and IBOA monomers.

#### 3.1.2. Structural Insights from Adsorption

**Figure 3:**
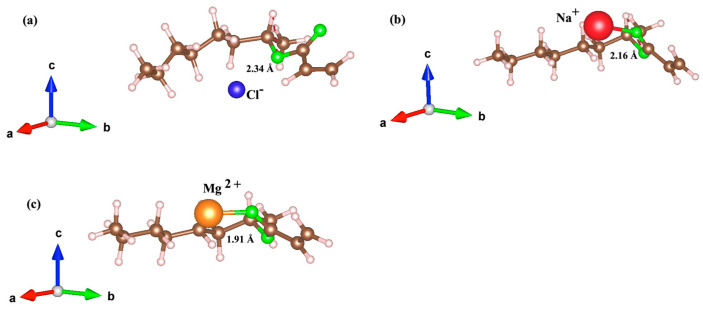
Ground relaxed structures of ions adsorbed on 2-OA monomers: (**a**) Cl^−^, (**b**) Na^+^, and (**c**) Mg^2+^. The corresponding nearest neighbor distances to the monomer with the input ion at position V are also included and given in numbers. The green, brown, and white spheres represent oxygen, carbon, and hydrogen atoms, respectively, while the blue, red, and orange spheres correspond to Cl^−^, Na^+^, and Mg^2+^ ions, respectively.

We next analyzed the bond structures and quantum descriptors to elucidate the underlying mechanisms driving the adsorption process of ions on the two monomers. We then focused on the lowest adsorption energy states for the ions on the two polymers. The results for the 2-OA polymer, specifically for the lowest energy configuration (site V), are presented in Figure 3.

The configuration with the lowest adsorption energy for Cl^−^ ions on the 2-OA monomer is relaxed slightly distant from the acrylate group; however, the Mg^2+^ and Na^+^ ions are relatively closer to the acrylate group due to stronger electrostatic attraction. The 2-OA monomer bends around the Cl^−^ ion effectively encapsulating it due to the strong electrostatic interactions with the carbonyl oxygen atoms, as shown in Figure 3a. This geometric configuration suggests that the ion is well separated from the monomer backbone, avoiding direct covalent bonding. Due to the electrostatic repulsion between the negatively charged Cl^−^ ion and surrounding electron-rich regions, the Cl^−^ ion is relaxed at an approximate separation distance of 2.34 Å from the H atom. On the other hand, the Na^+^ ion prefers to be adsorbed closer to acrylate, interacting with the electronegative oxygen atoms of the group. The relative size of the Na^+^ ion is larger compared to the Cl^−^ one, a fact that indicates a stronger interaction and potentially higher coordination with the monomer functional groups. Consequently, the neighbor distance of the Na^+^ ion is approximately 2.16 Å, which is shorter than that of the Cl^−^ ion. The magnesium Mg^2+^ adsorbed on 2-OA is shown in Figure 3c. The Mg^2+^ ion with a +2 charge forms stronger interactions with the acrylate group than the monovalent Na^+^ or Cl^−^ ions. In fact, the shorter bond distance with 1.91 Å and the lower adsorption energy suggest stronger electrostatic attractions.

**Figure 4:**
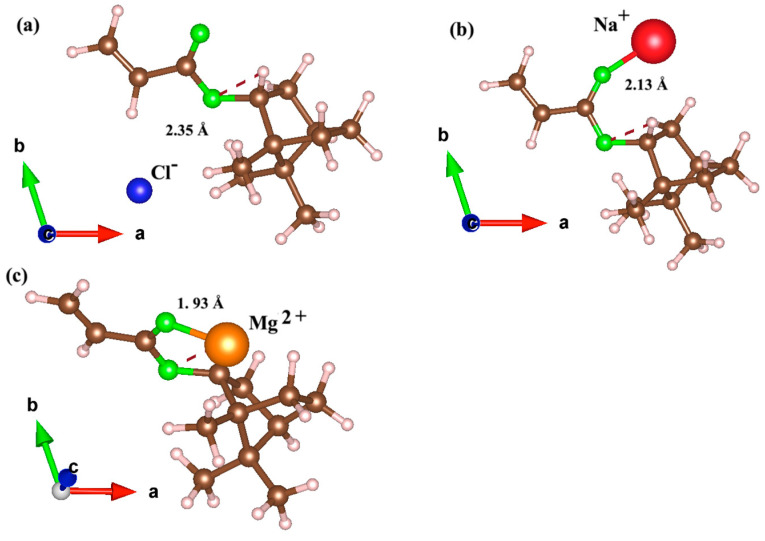
Ground relaxed structures of several ions adsorbed on IBOA monomers: (**a**) Cl^−^, (**b**) Na^+^, and (**c**) Mg^2+^. The corresponding nearest neighbor distances to the monomer are also included and given in numbers. The green, brown, and white spheres represent oxygen, carbon, and hydrogen atoms, respectively, while the blue, red, and orange spheres correspond to Cl^−^, Na^+^, and Mg^2+^ ions, respectively.

The calculated bond distances for ions on the IBOA monomer are shown Figure 4, specifically for the lowest energy configurations of the Cl^−^ ion at site III, and the Mg^2+^ and Na^+^ ions at site IV. Figure 4 shows the adsorption configurations for Cl^−^, Na^+^, and Mg^2+^ ions on the IBOA polymer surface. Panel (a) shows the Cl^−^ ion adsorbed at site III, with the nearest neighboring atom marked; panel (b) displays the Na^+^ ion at site IV; and panel (c) shows the Mg^2+^ ion at site IV. Each ion is visualized with its relative size and spatial orientation within the monomer, following its distinct interactions based on ionic charge and adsorption site. Similar to the behavior observed in 2-OA, the configuration with the lowest adsorption energy for Cl^−^ is relatively distant from the acrylate group, whereas for Mg^2+^ and Na^+^ it is located much closer to the acrylate group. The bond length of Cl^−^ is explained by a weaker interaction with the acrylate group, primarily due to its negative charge, which limits its affinity for the electron-rich oxygen atoms within this group. Instead, the Cl^−^ ions prefer more neutral or less electron-dense regions, as the electrostatic repulsion between the negatively charged Cl^−^ ion and the oxygen atoms reduces the possibility of close interactions. In contrast, both Mg^2+^ and Na^+^ ions exhibit a clear preference for adsorption sites near the oxygen atoms within the acrylate group, and they achieve their lowest adsorption energies in these positions. The Mg^2+^ ion, with the shortest bond distance of 1.93 Å, and Na^+^, with a bond distance of 2.13 Å, display strong electrostatic attractions to the negatively charged oxygen atoms, and accommodate in a more stable adsorption state. The high electron density around these oxygen atoms provides ideal sites for the adsorption of these cations, enabling stronger interactions and reduced adsorption energies. Overall, in both 2-OA and IBOA, Mg^2+^ ions are positioned approximately 2 Å away, while Cl^−^ and Na^+^ ions are positioned more than 2 Å away. This indicates that the adsorption mechanism seems driven by electrostatic interactions rather than chemical bonds. Therefore, the type of adsorption in both monomers can be mainly classified as physical adsorption.

Electrostatic interactions dominate the ion adsorption process, making the desorption process rapid and reversible depending on the ion energies due to temperature. In particular, as the Cl^−^ ion exhibits a relatively lower adsorption energy of 0.5 eV compared to Na^+^ and Mg^2+^ ions, at room temperature the Cl^−^ ion could desorb from monomers and diffuse across polymers and copolymers. Other organic molecules involving Cl and =O groups are used for adsorption to electrodes [39,40]; however, the bond is very different from the one of Cl^−^ ion to 2-OA and IBOA monomers.

#### 3.1.3. Quantum Descriptors

Table 1 collects several quantum descriptors for each ion on the 2-OA monomer, such as ionization energy (*I*), electron affinity (*A*), chemical potential (μ), hardness (η), and electrophilicity index (ω). The ionization potential (*I*) values for the Cl^−^, Na^+^, and Mg^2+^ ions are 0.92, 7.26, and 12.10, respectively. These values reflect the energy required to remove an electron from each ion, with higher ionization potentials indicating a greater tendency to retain electrons, and thus greater stability. The Mg^2+^ ion, being in a stable doubly charged state with a high ionization energy required to remove additional electrons, is the most stable among the three ions. In contrast, Cl^−^, as a stable anion with a complete electron shell, favors electrostatic interactions rather than covalent bond formation, given its stability in retaining its extra electron. Moreover, as shown in Table 1, the electrophilicity index (*ω*) of Cl^−^, Na^+^, and Mg^2+^ ions is 0.09, 39.40, and 40.90, respectively. An ion with a high electrophilicity index tends to form bonds with other molecules because this high index indicates a strong capacity for accepting electrons. Therefore, these results indicate that the Mg^2+^ and Na^+^ ions have a high tendency to form chemical bonds and are likely to engage in electron exchange rather than to hop between stable states in monomers. The relatively short bond lengths observed, along with the lower adsorption energies compared to the Cl^−^ ion, support this conclusion. For the Cl^−^ ion, the *ω* is notably low, suggesting that it tends to avoid bonding and instead engages primarily in electrostatic interactions hopping from site to site around the monomer and polymers, a finding that is further supported by the observed bending of the 2-OA monomer around the Cl^−^ ion.

Overall, the Mg^2+^ ions show the strongest adsorption, with the shortest distance to the nearest neighbor and the highest quantum descriptor values, typical of stronger interactions. The Na^+^ ion also exhibits significant adsorption strength, while the Cl^−^ ion shows the weakest interaction among the three ions. This data suggests that the charge and size of the ion play critical roles in the adsorption behavior and the resulting electronic properties of the system.

Table 2 collects several quantum descriptors for each ion on the IBOA monomer, including ionization energy (*I*), electron affinity (*A*), chemical potential (*μ*), hardness (*η*), and electrophilicity index (ω). The findings show that Mg^2+^ has the shortest nearest-neighbor distance at 1.93 Å and suggest a stronger interaction with the polymer compared to Cl^−^ and Na^+^. The ionization potential (*I*) values for Cl^−^, Na^+^, and Mg^2+^ ions are listed as 1.05, 8.65, and 11.98, respectively, in Table 2. Therefore, the Mg^2+^ ion has the highest values for both ionization energy and electron affinity, which is correlated with its greater chemical potential and electrophilicity and shows a more pronounced and localized bonding interaction within the polymer. In contrast, the relatively low ionization potential of Cl^−^ implies that it requires lower energy to lose its outermost electron, and thus it favors electrostatic interactions over covalent bond formation. The values for electrophilicity index (ω) of Cl^−^, Na^+^, and Mg^2+^ ions are 0.15, 12.0, and 37.7, respectively. The high ω values of Mg^2+^ and Na^+^ ions suggest a greater trend to form bonds by accepting electrons. The distinct quantum descriptors among the ions highlight differences in their chemical reactivities and stability within adsorption sites. The Cl^−^ ion, for example, displays lower hardness and electrophilicity, which implies a more flexible and less reactive interaction than that of the divalent Mg^2+^ ion. The Mg^2+^ ion has a high electrophilicity index and suggests a more stable and less reactive arrangement in similar conditions. These differences in quantum descriptors emphasize the unique adsorption behaviors of each ion on the IBOA monomer, shaped by their electronic characteristics and preferred adsorption sites.

### 3.2. Combining 2-OA and IBOA in Dimers

**Figure 5:**
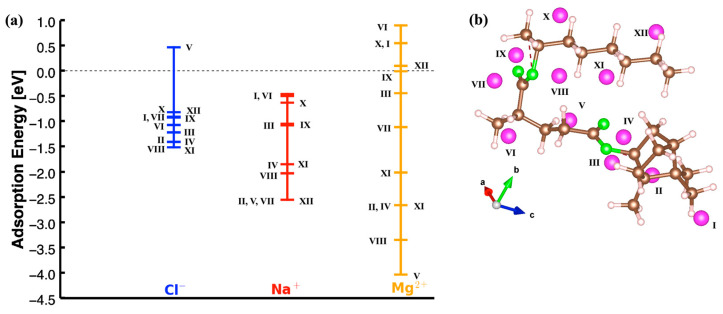
(**a**) Distribution of adsorption energy for Cl^−^, Na^+^, and Mg^2+^ ions across different positions on the dimer of 2-OA and IBOA monomers. (**b**) Adsorption configurations of the dimer 2-OA and IBOA with different sites. The pink, green, brown, and white spheres represent adsorbed ions (Cl^−^, Na^+^, Mg^2+^) given by roman numbers and the oxygen, carbon, and hydrogen atoms, respectively.

To further investigate the combined effect of the 2-OA and IBOA monomers, a dimer was built, and Cl^−^, Mg^2+^, and Na^+^ ions were placed at various positions near to their surface to calculate their adsorption energies, as shown in Figure 5. For the Cl^−^ ions, the adsorption energies are relatively high, with most of the site values clustered around the range from −0.5 eV to −1.5 eV levels, due to their relatively weaker interactions with the dimer. This trend agrees with the results in Figure 2 for the 2-OA and IBOA monomers, where the Cl^−^ ions also shows low adsorption energies and limited affinity for the acrylate group. In the dimer, the Cl^−^ ion similarly avoids the high-density electron regions such as those near the oxygen atoms, and its adsorption occurs at more neutral sites. This behavior supports the conclusion that Cl^−^ ions predominantly engage in weaker, electrostatic interactions, and remain positioned at a relatively distant point from the electron-rich acrylate groups.

The Na^+^ ion shows moderate adsorption energies, with values generally ranging from −0.5 to −2.5 eV. This intermediate range suggests that Na^+^ ions experience stronger interactions with the dimer compared to Cl^−^, but not as strong as Mg^2+^. In fact, the Mg^2+^ ions exhibit the strongest adsorption among the ions, with adsorption energies ranging from approximately 1.0 eV to −4.0 eV, with a wide range of interactions with the dimer surface. The adsorption sites for the Mg^2+^ ions are predominantly located near the acrylate oxygen atoms of both the 2-OA and IBOA segments, similar to the trend observed in the previous separate analyses for each monomer. The high adsorption energy and close proximity to the acrylate group suggest that Mg^2+^ forms the most stable interactions, which are likely facilitated by its high charge density and the strong electrostatic attraction to the electron-rich oxygen atoms. This positioning is in line with the previous findings where Mg^2+^ consistently showed a strong preference for acrylate sites due to the favorable electrostatic interactions. The adsorption behavior at site V differs significantly depending on the type of ion placed there. When the Cl^−^ ion is at site V, it exhibits a positive adsorption energy, which indicates an energetically unfavorable interaction. In contrast, when Mg^2+^ or Na^+^ ions are placed at the same site, they exhibit negative adsorption energies, with this site representing the lowest-energy configuration for both cations. This discrepancy is likely due to the electrostatic nature of site V and how it interacts differently with positively and negatively charged ions. The difference in adsorption energies can be attributed to the surrounding electrostatic environment at site V. Site V is in a region that is electron-deficient or surrounded by partially negative charges, so it is highly favorable for the positively charged Mg^2+^ and Na^+^ ions, which are attracted to regions where they can interact with negatively charged or electron-dense groups. This attraction reduces the adsorption energy, and results in a stable, low-energy configuration for Mg^2+^ and Na^+^. In contrast, when the Cl^−^ ion is placed at site V, the electron-rich surroundings or the presence of nearby positive charges creates a repulsive electrostatic interaction.

In comparison to the 2-OA and IBOA monomers, the dimer configuration enhances the availability of adsorption sites by combining the functional groups of both monomers with a broader range of electrostatic environments. The Cl^−^ ion continues to demonstrate the weakest adsorption and avoids the acrylate groups, while the Na^+^ and Mg^2+^ ions prefer to be adsorbed near oxygen-rich sites, with the Mg^2+^ showing the strongest binding affinity. This trend in the adsorption energies (E_ads_^Mg2+^ > E_ads_^Na+^ > E_ads_^Cl−^) remains consistent, and it shows that the dimer configuration does not significantly modify the inherent adsorption preferences of each ion on monomers but provides a larger and complex surface for ion interactions. This study underscores the role of ion charge and electron density distribution in dictating their adsorption behavior, with the Mg^2+^ ions benefiting most from the combined acrylate groups in the dimer structure, thereby achieving the most stable adsorption configurations.

For further analysis, the relevant quantum descriptors have also been calculated in the dimer, as included in Table 3. The quantum descriptor results for the dimer composed of 2-OA and IBOA monomers agree well with the trends observed in the 2-OA and IBOA monomers. The Mg^2+^ ion displays the highest values for stability-related descriptors, as there is strong adsorption and interaction, followed by Na^+^ with moderate descriptor values. In contrast, the Cl^−^ ions have comparatively lower descriptor values, because of their weaker adsorption. These results suggest that the combined dimer retains the electronic and stability characteristics seen in the individual monomers, with trends in quantum descriptors agreeing with the influence of ionic charge and electrostatic compatibility on adsorption behavior. No significant differences in adsorption energies or quantum descriptor values were observed when dimer was formed from either 2-OA or IBOA monomers. Consequently, the electronic properties and electrostatic potential characteristics of the AB dimer were herein excluded from further consideration.

### 3.3. Electronic Properties

**Figure 6:**
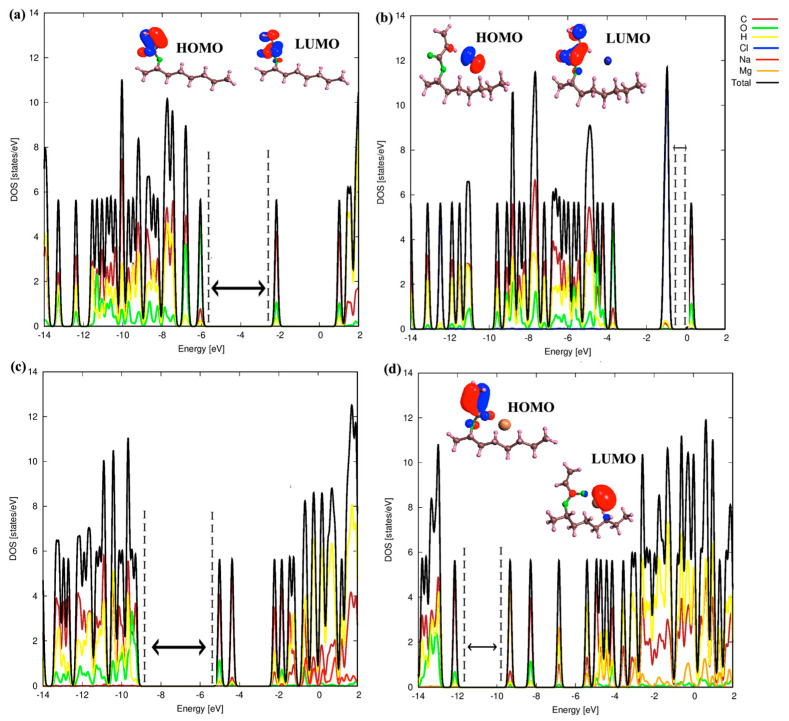
DOS of pure and ion adsorbed 2-OA monomers. The dashed lines define a region between the HOMO and LUMO levels. (**a**) DOS of pure 2-OA monomer. (**b**) DOS of Cl^−^ adsorbed 2-OA monomer. (**c**) DOS of Mg^2+^ adsorbed 2-OA monomer. (**d**) DOS of Na^+^ adsorbed 2-OA monomer.

We next focused on the electronic properties of 2-OA and IBOA monomers. In this context, the density of states for both pure and ion-adsorbed 2-OA and IBOA monomers were calculated. Figure 6 displays the density of states (DOS), highest occupied molecular orbitals (HOMO) and lowest unoccupied molecular orbital (LUMO) levels for the pure 2-OA monomer and with different ions (Cl^−^, Na^+^, Mg^2+^) adsorbed, illustrating how each ion modifies the electronic structure of 2-OA.

The 2-OA monomer exhibits a relatively wide HOMO-LUMO gap, which is related to its intrinsic electronic stability. Furthermore, the HOMO and LUMO levels are localized around the acrylate group, and the DOS shows that the contribution to the electronic states are mainly from O and C atoms, respectively. When the Cl^−^ ion is adsorbed onto 2-OA, the energy levels rise significantly, and they show a much smaller HOMO-LUMO gap because the Cl^−^ levels are in the middle gap of the 2-OA monomer. Moreover, this suggests that the Cl^−^ ion has a relatively weak interaction with 2-OA, a fact that agrees with the low adsorption energy and the quantum descriptor values. The Cl^−^ ion becomes the HOMO level of the whole system. The Na^+^ ion adsorbed on 2-OA is lowering the electronic levels compared to the pure 2-OA monomer indicating stabilization. This shift suggests that Na^+^ ion interacts with the 2-OA monomer, being stronger than the Cl^−^ ion. Moreover, the HOMO-LUMO gap can be compared to the one in the 2-OA monomer, having a similar character in the DOS mainly from O and C atoms, indicating that Na^+^ interacts with electron-dense regions in 2-OA, and that the Na levels are higher in the conduction band. This interaction aligns with the Na^+^ ion having intermediate adsorption energy and quantum descriptor values, with a stable adsorption, while inducing moderate changes in the electronic structure of 2-OA. The adsorption of the Mg^2+^ ion significantly modifies both the HOMO and LUMO energy levels compared to the 2-OA monomer. There is a large negative shift that indicates that the Mg^2+^ ion stabilizes the 2-OA structure through substantial electron transfer, in agreement with the high electrophilicity index and strong quantum descriptor values of Mg^2+^ given in Table 1. In addition, we observe a narrower HOMO-LUMO gap compared to the pure 2-OA monomer because the Mg^2+^ level is in the middle of the gap, a result of the strong ion–molecule interactions which enhance the stability of the Mg^2+^ ion adsorbed on 2-OA monomer. These interactions suggest that Mg^2+^ forms a stable, low-energy adsorption configuration, significantly influencing the electronic structure of 2-OA.

**Figure 7:**
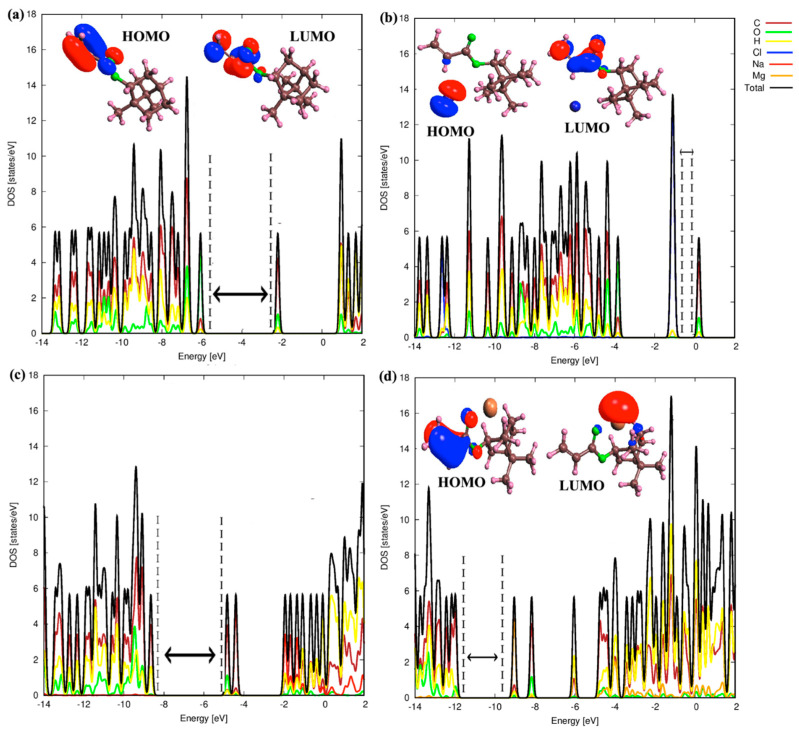
DOS of pure and ion adsorbed IBOA monomers. The dashed lines define a region between the HOMO and LUMO levels. (**a**) DOS of pure IBOA monomer. (**b**) DOS of Cl^−^ adsorbed IBOA monomer. (**c**) DOS of Mg^2+^ adsorbed IBOA monomer. (**d**) DOS of Na^+^ adsorbed IBOA monomer.

Figure 7 displays the DOS and HOMO-LUMO levels for the pure IBOA monomer and with different ions (Cl^−^, Na^+^, Mg^2+^) adsorbed. The pure IBOA monomer has similar HOMO and LUMO energy levels as the pure 2-OA monomer and with the same contribution of C and O atoms. For the Cl^−^ ion adsorbed on IBOA monomer, the Cl^−^ ion states in the middle become the combined HOMO level, without causing significant changes to the overall DOS pattern of the other atoms. This indicates that the Cl^−^ ion does not significantly alter the electronic structure of IBOA and remains weakly adsorbed through non-covalent interactions. Moreover, the energy levels increase, and the HOMO-LUMO gap becomes noticeably smaller after the adsorption of the Cl^−^ ion, resulting in weaker bonding interactions. This is consistent with its low electrophilicity and its tendency to avoid strong bonding with electron-dense areas in IBOA. When the Na^+^ ion is adsorbed, the HOMO and LUMO levels are lowered compared to the pure IBOA monomer, but the reduction is less pronounced than in the Mg^2+^ ion adsorbed case, reflecting weaker stabilization. This reduction also suggests that Na^+^ has a moderate interaction strength with IBOA, stronger than Cl^−^ but weaker than Mg^2+^. This result is consistent with the quantum descriptors for Na^+^, which demonstrate moderate stability and adsorption energy, contributing to a stable, although less intense interaction compared to Mg^2+^.

### 3.4. Electrostatic Potential

**Figure 8:**
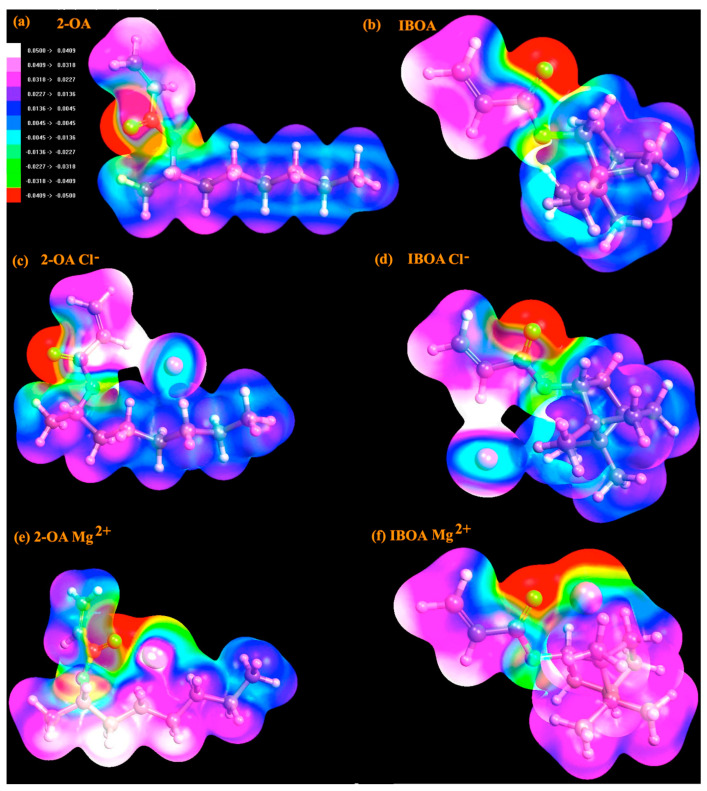
Electrostatic potential maps of ions adsorbed on 2-OA and IBOA monomers: (**a**,**b**) 2-OA and IBOA monomer, (**c**,**d**) adsorbed Cl^−^ ion, and (**e**,**f**) adsorbed Mg^2+^ ion.

The study of electrostatic potential (ESP) supplies considerable information about the charge distribution/adsorption energy when an ion interacts with a monomer. Then, we can gain deeper insight into the electrostatic potential distribution on 2-OA and IBOA and obtain better understanding of the adsorption energies and preferred positions of Cl^−^, Mg^2+^, and Na^+^ ions. The ESP maps are obtained by the free software ArgusLab 4.0.1 [41] using Intermediate Neglect of Diatomic Differential Overlap Hamiltonian (INDO) [42,43,44]. ArgusLab uses semi-empirical methods that are today still widely used in important studies [45,46,47,48], as they provide detailed analyses of electrostatic potential surfaces. They are suitable for large-volume polymeric systems and offer in-depth surface analysis. We tested their accuracy in small systems, because we might use them in the future to perform calculations on large polymers. To ensure reliability, the optimized monomers structures including ions obtained from DFT calculations were used as input. Figure 8 shows the ESP maps of 2-OA and IBOA monomers, the electrostatic potential scale from 0.05 eV to −0.05 eV when going from white to red, following the side scale of colors. Note that negative and positive ions have been placed in the regions with the higher and lower values of the electrostatic potential, respectively, for the 2-OA and IBOA monomers.

Figure 8a–f, shows the electrostatic potential surfaces first around 2-OA and IBOA monomers, and then with the presence of Cl^−^ and Mg^2+^ ions. The negative (red regions) and the positive (white regions) zones are rich and deficient in electrons, respectively. The ESP map of the pure 2-OA and IBOA monomers shows a relatively homogeneous distribution of electrostatic potential, except for the acrylate group, which exhibits significant negative potential regions concentrated around it. Particularly, the relatively positive and homogeneous electrostatic potential outside the acrylate group explains why the Cl^−^ ion exhibits a narrow adsorption energy range, because the electrostatic potential values outside the acrylate group fall within a similar range. Consequently, as expected and shown in Figure 8c,d, the lowest adsorption energy position of the Cl^−^ ion is located away from the acrylate group, that now presents a negative electrostatic potential. There is a strong negative electrostatic potential around the acrylate group, and as shown in Figure 8e,f, the positively charged Mg^2+^ ion likely interacts ionically with the oxygen atoms settling near the acrylate group.

The ESP maps provide visual confirmation of the interactions between 2-OA and IBOA monomers with ions. The strength of adsorption correlates with ion charges (Cl^−^, Mg^2+^) and electrophilicity and dipole properties of monomers. Furthermore, the data obtained from the ESP maps for both the Cl^−^ ion and the Mg^2+^ ion are consistent with our adsorption energy calculations and quantum descriptor values, and they clearly explain the positions where the lowest adsorption energies are observed. The ESP map for the positively charged Na^+^ ion was not considered, as it is expected to yield similar results to the ESP map for the Mg^2+^ ion. These results reinforce the importance of charge distribution and molecular electronic properties in adsorption phenomena.

## 4. Conclusions

We have studied the adsorption behavior and electronic properties of 2-octyl acrylate (2-OA) and isobornyl acrylate (IBOA) monomers with Cl^−^, Na^+^, and Mg^2+^ ions using Density Functional Theory (DFT). The adsorption energy analysis revealed that Mg^2+^ ions consistently exhibit the strongest interactions in all systems due to their higher charge density, followed by Na^+^ and Cl^−^ ions. The energetically most favorable adsorption positions were identified for each ion on both monomers, with significant adsorption energies observed at specific sites, particularly around electron-rich oxygen atoms for cations (Na^+^, Mg^2+^) and general bindings positions around monomers for anions (Cl^−^).

In the 2-OA monomer, the lowest adsorption energies correspond to Na^+^ and Mg^2+^ with strong interactions near the acrylate groups. Cl^−^ ions, however, showed weaker and more evenly distributed adsorption energies across multiple positions, a fact that shows their tendency towards weaker electrostatic interactions. In IBOA, adsorption around the acrylate groups proved to be the most favorable for the Na^+^ and Mg^2+^ ions, with Mg^2+^ showing significantly higher adsorption energy due to its divalent nature. The specific structural configuration of the IBOA monomer, with its branched isobornyl group, contributes to this positional preference by offering localized electron densities. The adsorption behavior of the ions was further corroborated by electrostatic potential maps, which visually showed regions of high charge density as favorable adsorption sites. The study highlights that ion charge densities, molecular geometries, and site-specific electronic properties play a key role in determining ion adsorption efficiency. The performance of materials can be significantly modified by selecting specific adsorption sites and using ions with favorable charge densities. The results of this study provide a solid framework for addressing the chemical attack of ions on materials covered with acrylate compounds in harsh marine environments. Moreover, by adsorbing Cl^−^ the chemical surface eliminates the positive charges and becomes more negative, so living proteins would not attach easily to these polymer coatings, which could even serve to have antifouling properties.

## Figures and Tables

**Table 1:** Quantum descriptors (in eV) of ions on 2-OA at adsorption site V.

2-OA	*I*	*A*	μ	η	ω
Cl^−^	0.92	−0.28	−0.32	0.60	0.09
Na^+^	7.26	6.13	−6.70	0.57	39.40
Mg^2+^	12.10	9.32	−10.70	1.40	40.90

**Table 2:** Quantum descriptors (in eV) of ions on IBOA of the adsorption for Cl^−^ at site III and for Na^+^ and Mg^2+^ ions at site IV.

IBOA	*I*	*A*	μ	η	ω
Cl^−^	1.05	−0.2	−0.43	0.63	0.15
Na^+^	8.65	4.85	−6.75	1.9	12.00
Mg^2+^	11.98	9.05	−10.52	1.47	37.65

**Table 3:** Quantum descriptors (in eV) of ions on 2-OA and IBOA dimer at adsorption site VIII for Cl^−^ and V for Na^+^ and Mg^2+^ ions.

Dimer	*I*	*A*	μ	η	ω
Cl^−^	1.61	−1.25	−0.18	1.43	0.01
Na^+^	8.30	3.80	−6.05	2.25	8.13
Mg^2+^	9.76	7.23	−8.50	1.27	28.45

## Data Availability

The data presented in this study are available on request from the corresponding author due to privacy reasons.
